# Supplementary material for: Divergent roles of macrophage subsets, FoxP3, and IL-17A in HSV-1–induced CNS pathology
Source: PLoS Pathog. 2025 Nov 17;21(11):e1013696. doi: 10.1371/journal.ppat.1013696 (PMC12633891; doi:10.1371/journal.ppat.1013696)
Supplement: S1 Data — (PDF) [file ppat.1013696.s001.pdf]

CD4+ T cells

	Exp01	Exp02	Exp03

HSV-IL-2

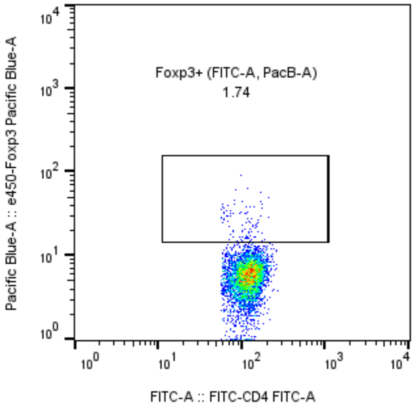

1.74 %

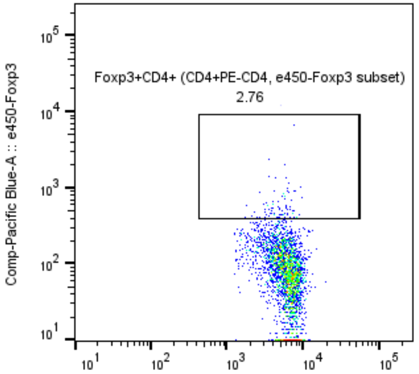

2.78 %

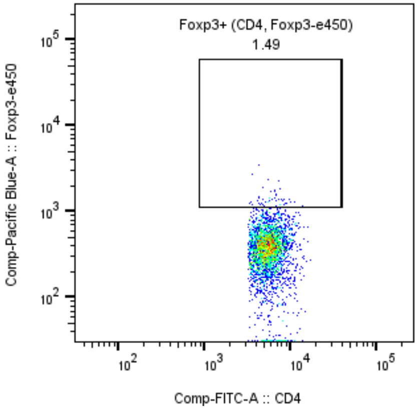

1.49 %

HSV-1

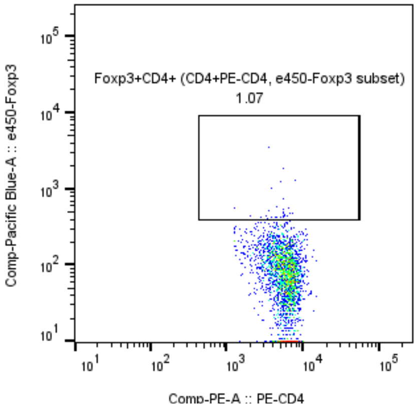

1.07 %

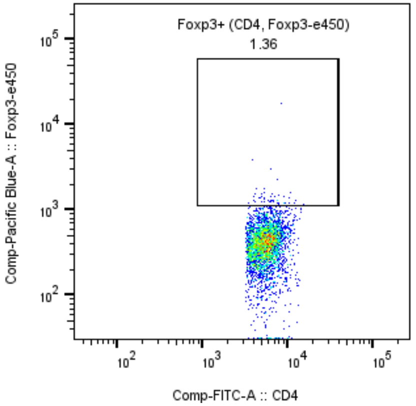

1.36 %

CD8+ T cells

HSV-IL-2

Exp01

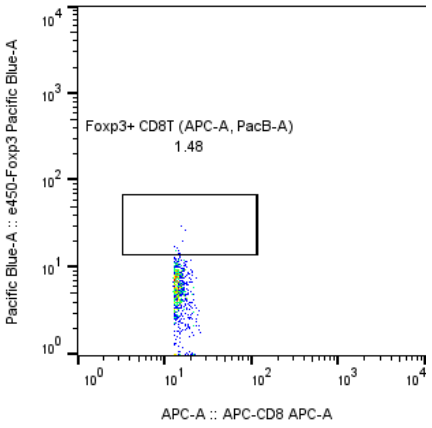

1.48 %

Exp02

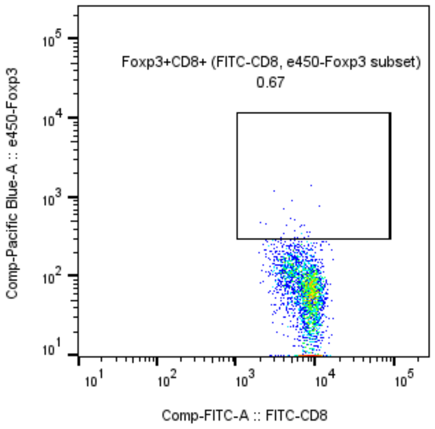

0.57 %

Exp03

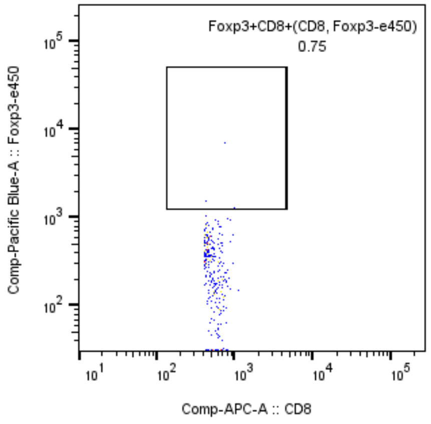

0.75 %

HSV-1

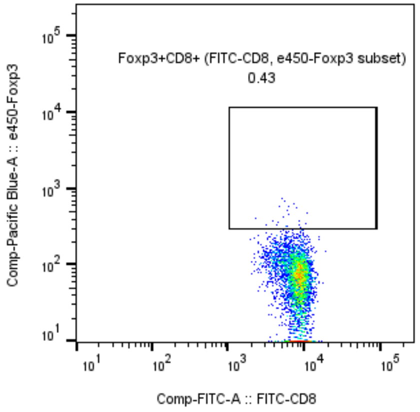

0.43 %

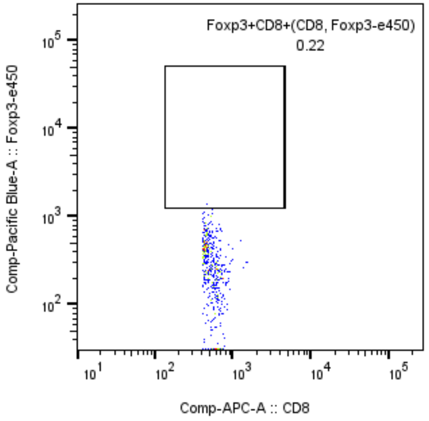

0.22 %

	CD8+ T cells		CD4+ T cells	
	HSV-IL-2	HSV-1	HSV-IL-2	HSV-1
Exp01	1.48 %		1.74 %	
Exp02	0.57 %	0.43 %	2.78 %	1.07 %
Exp03	0.75 %	0.22 %	1.49 %	1.36 %

Raw data for Fig. 2 (Frequencies of $CD4^+$ and $CD8^+$ T cells following infection with HSV-IL-2 or HSV-1). Percentages of $CD4^+$ and $CD8^+$ T cells were measured in independent experiments (Exp01–Exp03) following infection with recombinant HSV expressing IL-2 (HSV-IL-2) or wild-type HSV-1. Each point represents an individual experiment, and the graphs indicate the percentage of T cells that are positive under each condition.

		No. of animals	No.of Animals with Demylination	No.of sections	No. of demilinations	animal 1	animal 2	animal 3	animal 4
[illegible] 3-DTR,VIL2-inf	Brain	5	4	80	10	1	4	3	
	SC	5	4	80	12	4	1	4	3
	ON	5	2	80	5	2	3		
3-DTR,VIL2-inf,Mf-dpl	Brain	5	5	80	28	5	7	6	5
	SC	5	5	80	22	4	6	3	4
	ON	5	5	80	10	2	1	2	3
DTR-Foxp3-Mf-dep,VIL2-	Brain	4	3	80	7	2	2	3	
	SC	4	4	80	10	3	2	4	1
	ON	4	2	80	5	2	3		
C57,Fox-DTR,VIL4-inf	Brain	5	0	80	0				
	SC	5	0	80	0				
	ON	5	0	80	0				
DTR,FOXP3-dpl,VIL2-	Brain	5	0	80	0	0	0	0	
	SC	5	0	80	0	0	0	0	
	ON	5	0	80	0	0	0	0	
C57,Fox-DTR,MCK-inf,Mf-dpl	Brain	2	2	40	5	2	4		
	SC	2	2	40	7	3	4		
	ON	2	2	40	6	3	3		
DTR,MCK-inf,Mf&Fox P3-dpl	Brain	3	0	75	0				
	SC	3	0	75	0				
	ON	3	0	75	0				
C57,IL17a-ko,VIL2-inf	Brain	3	0	80	0	0	0	0	
	SC	3	0	80	0	0	0	0	
	ON	3	0	80	0	0	0	0	
C57,IL17a-ko,VIL2-inf,Mf_dpl	Brain	3	0	80	0	0	0	0	
	SC	3	0	80	0	0	0	0	
	ON	3	0	80	0	0	0	0	
C57,IL17r-ko,VIL2-inf,Mf_dpl	Brain	3	3	80	13	4	5	4	
	SC	3	3	80	19	6	6	7	
	ON	3	3	80	6	2	3	1	

ko,VIL2-inf,Mock_dpl	Brain	5	5	80	18	2	3	5	4
	SC	5	5	80	18	2	2	5	5
	ON	5	5	80	6	1	2	1	1

C57, VIL2-inf	Brain	5	5	70	8	2	1	2	2
	SC	5	5	70	10	3	2	2	2
	ON	5	5	75	11	2	3	1	3

Original data for Fig. 4 (quantification of demyelination in the CNS of experimenta

animal 5	Mean	SEM	StD		cal. No. Animals W Demy/ No. of animals	cal. No. Demy/ No. of section	size of demy.
2	2.213364	0.645497	1.290994		80%	13%	Brain
	2.632148	0.707107	1.414214		80%	15%	SC
	2.44949	0.5	0.707107		40%	6%	ON
5	5.546664	0.4	0.894427		100%	35%	Brain
5	4.282255	0.509902	1.140175		100%	28%	SC
2	1.888175	0.316228	0.707107		100%	13%	ON
	2.289428	0.333333	0.57735		75%	9%	Brain
	2.213364	0.645497	1.290994		100%	13%	SC
	2.44949	0.5	0.707107		50%	6%	ON
	#NUM!	#DIV/0!	#DIV/0!		0%	0%	Brain
	#NUM!	#DIV/0!	#DIV/0!		0%	0%	SC
			#DIV/0!		0%	0%	ON
			0		0%	0%	Brain
			0		0%	0%	SC
			0		0%	0%	ON
	2.828427	1	1.414214		100%	13%	Brain
	3.464102	0.5	0.707107		100%	18%	SC
	3	0	0		100%	15%	ON
	#NUM!	#DIV/0!	#DIV/0!		0%	0%	Brain
	#NUM!	#DIV/0!	#DIV/0!		0%	0%	SC
	#NUM!	#DIV/0!	#DIV/0!		0%	0%	ON

			0		0%	0%	Brain
			0		0%	0%	SC
			0		0%	0%	ON
	#NUM!	0	0		0%	0%	Brain
	#NUM!	0	0		0%	0%	SC
	#NUM!	0	0		0%	0%	ON
	4.308869	0.333333	0.57735		100%	16%	Brain
	6.31636	0.333333	0.57735		100%	24%	SC
	1.817121	0.57735	1		100%	8%	ON

4	3.437544	0.509902	1.140175	100%	23%	Brain
4	3.314454	0.678233	1.516575	100%	23%	SC
1	1.148698	0.2	0.447214	100%	8%	ON

1	1.515717	0.244949	0.547723	100%	11%	Brain
1	1.888175	0.316228	0.707107	100%	14%	SC
2	2.047673	0.374166	0.83666	100%	15%	ON

l mouse groups). Table summarizing the extent of demyelination observed in the brain, spinal cc

animal 1	animal 2	animal 3	animal 4	animal 5	Mean	SEM	StD
218	185	122		136	160.8354	22.17121	44.34242
302	286	263	231		269.1443	15.40833	30.81666
83	96				89.26365	6.5	9.192388
242	302	206	178	213	224.5421	21.07226	47.119
293	308	293	254	199	266.1917	19.74487	44.15088
67	75	64	58	89	69.82755	5.353504	11.9708
123	142	225			157.8067	31.31737	54.24328
312	269	231	109		214.4057	43.67183	87.34367
64	59				61.44917	2.5	3.535534
					#NUM!	#DIV/0!	#DIV/0!
					#NUM!	#DIV/0!	#DIV/0!
				0			#DIV/0!
0	0	0					0
0	0	0					0
0	0	0					0
263	179				216.9723	42	59.39697
226	316				267.2377	45	63.63961
41	49				44.82187	4	5.656854
					#NUM!	#DIV/0!	#DIV/0!
					#NUM!	#DIV/0!	#DIV/0!
					#NUM!	#DIV/0!	#DIV/0!

0	0	0					0
0	0	0					0
0	0	0					0
0	0	0			#NUM!	0	0
0	0	0			#NUM!	0	0
0	0	0			#NUM!	0	0
113	93	102			102.342	5.783117	10.01665
188	192	210			196.4383	6.765928	11.71893
49	64	84			64.104	10.13794	17.55942

163	124	84	105	86	108.9219		32.60828
171	126	205	171	165	165.6228	12.56821	28.10338
51	65	41	52	39	48.76038		10.38268

119	98	96	92	128	105.6838	7.110556	15.89969
170	157	148	168	132	154.3472	6.9857	15.6205
54	43	52	42	45	46.95323	2.437212	5.449771

ord (SC), and optic nerve (ON) across various experimental groups. Each group consisted of *n* = 2

2–5 animals , with the number of demyelinated animals and the total number of sections analyze

d indicated. Individual animal values (animal 1–5) are shown, along with group means ± SEM. “N

o. of demyelination” refers to the total demyelinated lesions identified per animal across all sec

tions analyzed (typically 70–80 sections per region). Groups include C57BL/6 wild-type, FoxP3-D

)TR, IL17a$^{-/-}$, VIL2-inf, MCK-inf, and combinations thereof with macrophage (Mf) or regulatory T c

ell (FoxP3$^+$) depletion as indicated.
