# Supplementary material for: Divergent roles of macrophage subsets, FoxP3, and IL-17A in HSV-1–induced CNS pathology
Source: PLoS Pathog. 2025 Nov 17;21(11):e1013696. doi: 10.1371/journal.ppat.1013696 (PMC12633891; doi:10.1371/journal.ppat.1013696)
Supplement: Attachment — Submitted filename: Response (10-17-2025)PP xxx.doc [file ppat.1013696.s003.doc]

**RE**: PPATHOGENS-D-25-02100 Old Title (Title: Synergistic actions of M1 macrophages and TH17 in CNS demyelination); New Title (**Divergent roles of macrophage subsets, FoxP3, and IL-17A in HSV–1–induced CNS pathology).**

Dear Drs. Shukla, Neumann, Bhaduri-McIntosh, and Malim,

Below are our detailed responses to each reviewer's comments, outlining the changes we have made to the manuscript. Thank you for the opportunity to revise and resubmit our manuscript.

**Reviewer #1 comment**: In this well-designed and documented study, the authors investigated the roles of TH17, Treg, and macrophage subsets (M1 and M2) in murine models of CNS demyelination following HSV-1 ocular infection. The study provides novel observations concerning the contributions of immune signaling networks, particularly those involving IL-17A and macrophage polarization, to demyelinating pathology. The study highlights a critical role for M1 macrophages in promoting CNS demyelination, driving TH17 activation, and influencing FoxP3⁺ Treg dynamics. These findings are novel and highly relevant to the field. The study is generally well documented, however, in my opinion, the clarity of the presentation needs improvement in a few areas.

**Reviewer #2 Comment**: The manuscript titled “Synergistic actions of M1 macrophages and TH17 in CNS demyelination” by Jaggi and co-workers explores the interplay between macrophages, FoxP3+ regulatory T cells, and IL-17 in CNS demyelination following ocular infection with HSV-1. The authors demonstrate that infection with an IL-2-expressing HSV-1 strain leads to CNS demyelination in mice depleted of both macrophages and FoxP3+ cells, whereas WT HSV-1 infection does not induce demyelination under the same conditions. Interestingly, macrophage depletion alone during WT HSV-1 infection was sufficient to trigger demyelination, suggesting a protective role for macrophages in this context. To further investigate the contribution of macrophage subsets, the authors utilized M1- and M2-deficient mice. They report that the absence of M1 macrophages prevented demyelination, while M2-deficient mice exhibited CNS demyelination in the HSV-IL-2 strain, supporting a pathogenic role for M1 and a protective role for M2 macrophages. Additionally, depletion of macrophages in IL-17-deficient mice infected with HSV-IL-2, did not lead to demyelination, indicating that IL-17 contributes to demyelination in a macrophage-dependent manner and the absence of IL-17-macrophage is beneficial. Overall, the findings are novel and provide valuable insights into the distinct and non-redundant roles of macrophages, Th17, and FoxP3+ Tregs in modulating CNS pathology during HSV-1 infection. The study is well-conceived and contributes meaningfully to our understanding of neuroinflammatory mechanisms.

**Reviewer #3 Comment**: Summary: Multiple sclerosis is a major demyelinating neurologic disease that is associated with an immunopathologic origin. The Ghiasi laboratory has developed an intriguing mouse model for demyelinating neurologic diseases in which C57BL/6 mice are inoculated corneally with an IL-2-expressing recombinant HSV-1 (HSV-IL-2). These animals subsequently show demyelination in optic nerves, brain, and spinal cord. Because previous work has shown that depletion of macrophages induces demyelination in HSV-IL-2-infected mice, a series of studies were performed to assess further a role for macrophages in demyelination with emphasis on FoxP3 (T regulatory cells or Treg cells) and IL-17A participation. Results revealed (i) combined depletion of macrophages and FoxP3 triggered CNS demyelination in HSV-IL-2-infected mice but prevented demyelination in WT HSV-1-infected mice; (ii) depletion of macrophages in IL-17A-deficient mice did not restore CNS demyelination in HSV-IL-2-infected mice; and (iii) M1 macrophages are key drivers of plaque formation. The authors conclude that FoxP3, IL-17A, and macrophage subsets play distinct and non-redundant roles in modulating CNS demyelination during HSV-1 infection and suggest that targeting M1 macrophage activation might be a new therapeutic approach for limiting CNS demyelination.

Review: This investigation summarizes the findings of a series of relatively straightforward studies that add new and important knowledge regarding the relative roles of macrophages, FoxP3, and IL-17A during CNS demyelination using a well-established HSV-1 mouse model of the CNS demyelination. The experiments appear to be performed carefully with rigor and data are convincing. Although the manuscript is already strong in form and content, some attention to the following observations might further strengthen the manuscript.

**Comment 1**. The Title is far too brief and uninformative and needs to be revised to inform potential readers that the investigation and conclusions as stated involve a mouse model of HSV-1 CNS demyelination.

RESPONSE. Agree. We changed the title to: Divergent roles of macrophage subsets, FoxP3, and IL-17A in HSV-1–induced CNS pathology.

**Comment 2**. Are paragraphs 2 and 3 of the Introduction section necessary? They summarize previous work on IL-12p35 and IL-12p40 as well as innate lymphoid cells that distract from the true focus of the studies summarized in the manuscript.

RESPONSE. Agree. We condensed paragraphs 2 and 3.

**Comment 3**. Although the inclusion of histopathologic figures in the Results section are convincing, the authors should provide quantification perhaps as percent animals showing demyelination versus total number of animals evaluated. Simply put, of the animals used per experimental group, how many were positive and negative for CNS demyelination? If 100%, then communicate this to the reader. Either provide this information in the Results section text or provide a table summarizing the results quantitatively.

RESPONSE. We added this information in the M&M section, lines 345-347.

**Comment 4**. Figure 4 is very blurry and difficult to read.

RESPONSE. Agree. Modified.

**Comment 5**. Is data on size and number of plaques (Fig 4) derived from the same experiment shown in Fig 1, or are these separate experiments?

RESPONSE. It is derived from the same experiment shown in Fig. 1.

**Comment 6**. The authors should caution against the statement that the KOS strain of HSV-1 is “avirulent” which means an absolute lack of disease/pathology. When compared with the McKrae strain, the KOS strain is weakly virulent.

RESPONSE. Agree. The word “avirulent” is changed to ‘weakly virulent’.

**Reviewer suggestion 1**: In the Demyelination morphometry section of the Materials and Methods section, the authors state that “The presence or absence of demyelination in the CNS of five infected mice per group was evaluated using luxol fast blue (LFB) staining as we described previously [14].”

It would seem important to provide here some additional information based on previous published work that LFB staining in the used mouse models at the studied time (14 days PI with virus) and in the studied CNS tissues LFB staining alone is sufficient to identify demyelinating lesions. Brief description of these lesions in the used models as far as myelin loss, axonal preservation and presence or absence of virus/viral products would be important for the better understanding of the model.

RESPONSE. Agree, and our focus of the study was to detect where myelin is lost or reduced; therefore, using LFB in our study is the norm to evaluate CNS demyelination.

**Reviewer suggestion 2**: Due to repetition of text, correction is needed to the sentence in lines 83—86, “Although, IL-12p70 is produced by a variety of immunocompetent cells, including monocytes, dendritic cells, neutrophils, and B cells [11, 12], in addition to macrophages IL-12p70 is produced by a variety of immunocompetent cells, including monocytes, dendritic cells, neutrophils, and B cells [11, 12],….

Reviewer #2: I have the following specific comments for the authors:

Line 83-86, delete the duplicated sentence “IL-12p70 is produced by a……….B cells”

RESPONSE. Agree. This line is now removed.

**Reviewer suggestion 3**: Fig 2A-B bar graphs, the X axis is labeled as HSV-IL-2, while the other bar is labeled as control. For clarity and consistency, I suggest relabeling control as WT HSV-1. Also, if available, it would strengthen the data presentation if authors include representative flow cytometry dot plots for the analyzed cell populations.

RESPONSE. Agree. Control is now called WT HSV-1, and a representative of FACS data is now included in the revised Fig. 2.

**Reviewer suggestion 4**: Line 179-182, the sentence seems incomplete; the authors should correct/reframe this sentence by citing ….…HSV-IL-2 infected mice (for 3 A-C) and …….WT HSV-1 infected mice for Fig 3D-F.

RESPONSE. Lines 164-168 are now revised.

**Reviewer suggestion 5**: Lines 182-183, the authors make a statement about a comparison between IL17A KO mice infected with HSV-IL-2 and WT HSV-1 strains. However, it appears that this data is missing. Fig. 3 only shows a comparison of IL-17 KO-macrophage-depleted mice with HSV-IL-2 infection with WT-HSV1-infected WT mice with macrophage depletion. Authors should verify if this is an error and show the data or correct the statement.

RESPONSE. This information was discussed in lines 156-157 of the results section. We did not discuss it in the current study since we published previously.

**Reviewer suggestion 6**: In Fig. 3, some of the panel labels (A-F) are on micrographs, while others are outside, depending on the size of the tissue section. It would be better if the authors keep these labels out of micrographs.

RESPONSE. Agree. Done.

**Reviewer suggestion 7**: Figure 4 (A-F) axis labels are tiny and unreadable. Can authors improve the visualization for better clarity?

RESPONSE. Figure 4 is now modified.

**Reviewer suggestion 8**: For Figs. 4A-F, the WT virus is simply labeled as HSV-1, whereas in all other figures, it is labeled as WT HSV-1. The authors may want to consider changing this to maintain consistency with the rest of the figures.

RESPONSE. Done.

**Reviewer suggestion 9**: For Fig. 4, the statistics are described in the results text but are not included in the figures. It would be easier for readers if they show statistics (either * or p values, as they did in Fig. 2), on figures/bars as well.

RESPONSE. The stats are now indicated in the legend to Fig. 4.

**Reviewer suggestion 10**: Lines 623-624, the figure legend statement is incomplete, and the mentions for the viral strains (HSV-1-IL-2 vs. WT HSV-1) are missing after the statements ……..ON, SC, and brain….from ……

RESPONSE. Done.

**Reviewer suggestion 11**: In Fig. 5, it appears that scale (black) bars are present on some micrographs but not all. For consistency and clarity, please ensure that scale bars are included on all images. Additionally, consider removing the magnification (X) from the figure legends and adding a scale bar to all micrograph panels, as scale bars provide a more precise and interpretable reference.

RESPONSE. Done.

**Reviewer #3:** Figure 4 is very blurry and difficult to read.

RESPONSE. Agree. It is now modified.

Thank you very much for allowing us to revise and resubmit our manuscript. We trust that we have satisfactorily addressed these helpful reviewers’ comments, and we have now made all the requested changes. New text in the body of the manuscript is printed in “Blue Arial” rather than “Black Arial”. We hope that the manuscript will now be found to be acceptable for publication in PLOS Pathogens.

Best Regards,

Homayon
